# The complete chloroplast genome of *Fraxinus malacophylla* (Oleaceae, Oleoideae)

**DOI:** 10.1080/23802359.2020.1827999

**Published:** 2020-10-21

**Authors:** Hua-Chao Duan, Xin-Hua Zheng, Yan-Yan Li, Shi-Min Li, Lan Ye, Hui-Zhu Jing, Qiong Dong

**Affiliations:** a Key Laboratory of National Forestry and Grassland Administration on Biodiversity Conservation in Southwest China, Southwest Forestry University, Kunming, China; b Forestry College of Southwest Forestry University, Kunming, China

**Keywords:** *Fraxinus malacophylla*, chloroplast genome, phylogeny

## Abstract

*Fraxinus malacophylla* is one of the commonly used ecological restoration tree species in rocky desertification areas. It has high medicinal and timber value. And has high marketization prospects. The complete chloroplast genome sequence of *F. malacophylla* was generated by de novo assembly using whole-genome next generation sequencing. The complete chloroplast genome of *F. malacophylla* was 155621 bp in total sequence length and divided into four distinct regions: large single copy region (86404 bp), small single copy region (17821 bp), and a pair of inverted repeat regions (25698 bp). The *F. malacophylla* chloroplast genome annotation predicted a total of 131 genes, consisting of 35 tRNA genes, 8 rRNA genes, and 88 protein-coding genes. Phylogenetic analysis with the reported chloroplast genomes revealed that *F. malacophylla* has most closely related to *F. excelsior*.

*Fraxinus malacophylla* is a deciduous tree, which mainly distributed in mountains secondary forest dominated by limestone in Yunnan and Guangxi, China (Huang et al. 2019). Its a commonly associated tree species for ecological restoration in rocky desertification areas (Huang and Chen 2014). *Fraxinus malacophylla* has high medicinal and timber value and usually be used to treat constipation, malaria, epilepsy and make furniture (Tan et al. 2013; Huang and Chen 2014). However, there are no researches about the *Fraxinus* chloroplast. Therefore, we first assembled the complete chloroplast genome of *F. malacophylla*, which provides a genomic resource and to clarify the phylogenetic relationship of this plant with other species in the Oleaceae family.

Leaf samples of *F. malacophylla* were collected from Kunming, Yunnan, China (geospatial coordinates: E102°46′40″, N25°03′07″, altitude: 1954 m). The voucher specimen is deposited at the Southwest Forestry University Herbarium (specimen number: SWFC0048721). The total DNA was isolated using the Plant Genomic DNA Kit. Then, the Illumina Hiseq X Ten platform was used to sequence the DNA (Sangon Biotech (Shanghai) Co. Shanghai, China). The complete cp genomes were assembled by SPAdes (Bankevich et al. 2012), Using GapFiller to add GAP to the contig obtained by stitching (Boetzer and Pirovano 2012), which compared with the chloroplast sequence of *F. xanthoxyloides* as a reference. PrInSeS-G was used for sequence correction to correct editing errors and missing insertion of small fragments in the process of splicing (Massouras et al. 2010). The genes in the chloroplast genome were predicted using Prokka (Seemann 2014) and corrected by Blast search. Simple sequence repeat (SSR) motifs were investigated using RepeatMasker (Altschul et al, 1997). The CpDNA sequence of *F. malacophylla* was submitted to GenBank (accession number: MT663306).

The chloroplast genome of *F. malacophylla* was a circular form of 1,55,621 bp in length, which was composed of four distinct regions such as large single copy (LSC) region of 86,404 bp, small single copy (SSC) region 17,821 bp and a pair of IRs regions of 25698. The overall GC content was 37.9% and the GC contents of the LSC, SSC, and IR regions are 35.9%, 32.1%, and 43.2%, respectively. The chloroplast genome contained a total of 133 genes, including 88 protein coding genes, 35 tRNA genes and 8 rRNA genes.

In order to understand the phylogenetic relationship between *F. malacophylla* and related species, the complete chloroplast genome sequences of 13 genera (28 species) were aligned by MAFFT (Katoh et al. 2002) and trimmed properly by trimAl (Capella-Gutierrez et al. 2009). The evolutionary history was inferred by using the Maximum Likelihood method based on the Tamura-Nei model in MEGA7.0 (Kumar et al. 2016). Bootstrap (BS) values were calculated from 1000 replicate analyses (Figure 1). As was expected, *F. malacophylla* was placed within Apiaceae tribe Fraxineae, and comprise a clade with *F. insularis*, *F. lanuginosa*, *F. xanthoxyloides* and *F. excelsior* subsp. greenmannii with 100% BS value.

**Figure 1.**
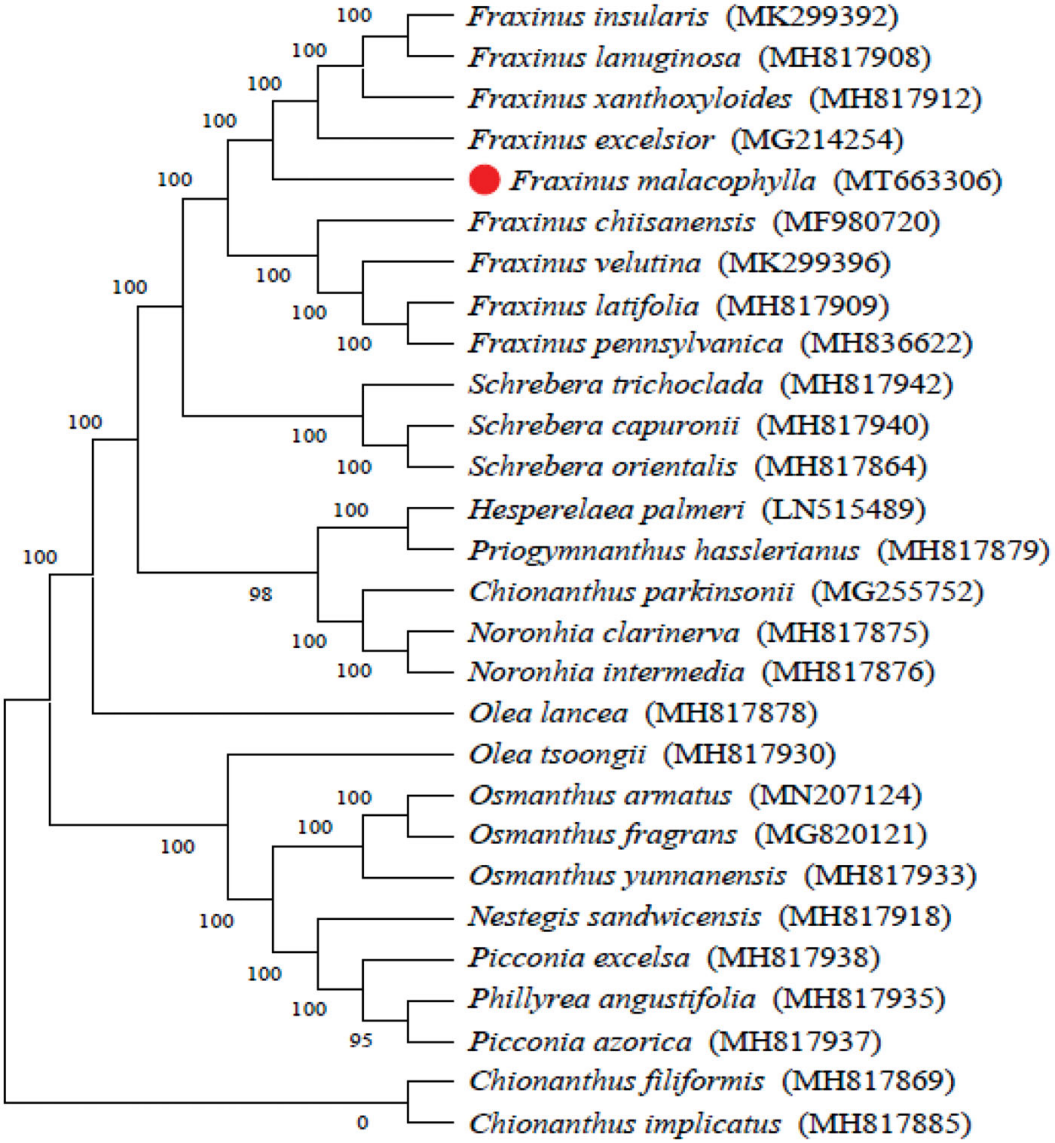
The maximum likelihood phylogenetic tree constructed from 28 species chloroplast genomes. The numbers at each node indicate bootstrap support.

## Data Availability

The data that support the findings of this study are openly available in [National Center for Biotechnology Information], [https://www.ncbi.nlm.nih.gov/], accession number [MT663306].
